# Deceptively simple … The “deception-general” ability and the need to put the liar under the spotlight

**DOI:** 10.3389/fnins.2013.00152

**Published:** 2013-08-29

**Authors:** Gordon R. T. Wright, Christopher J. Berry, Geoffrey Bird

**Affiliations:** ^1^Social Interaction Lab, Department of Psychological Sciences, Birkbeck, University of LondonLondon, UK; ^2^School of Psychology, Plymouth UniversityPlymouth, UK; ^3^MRC Social, Genetic and Developmental Psychiatry Centre, Institute of Psychiatry, King's College LondonLondon, UK; ^4^Institute of Cognitive Neuroscience, UCLLondon, UK

**Keywords:** “deception-general” ability, signal detection theory, deception, lying, social cognition

## Abstract

This Focused Review expands upon our original paper (You can't kid a kidder": Interaction between production and detection of deception in an interactive deception task. *Frontiers in Human Neuroscience*, 6:87). In that paper we introduced a new socially interactive, laboratory-based task, the Deceptive Interaction Task (DeceIT), and used it to measure individuals' ability to lie, their ability to detect the lies of others, and potential individual difference measures contributing to these abilities. We showed that the two skills were correlated; better liars made better lie detectors (a “deception general” ability) and this ability seemed to be independent of cognitive (IQ) and emotional (EQ) intelligence. Here, following the Focused Review format, we outline the method and results of the original paper and comment more on the value of lab-based experimental studies of deception, which have attracted criticism in recent years. While acknowledging that experimental paradigms may fail to recreate the full complexity and potential seriousness of real-world deceptive behavior, we suggest that lab-based deception paradigms can offer valuable insight into ecologically-valid deceptive behavior. The use of the DeceIT procedure enabled deception to be studied in an interactive setting, with motivated participants, and importantly allowed the study of both the liar and the lie detector within the same deceptive interaction. It is our thesis that by addressing deception more holistically—by bringing the liar into the “spotlight” which is typically trained exclusively on the lie detector—we may further enhance our understanding of deception.

## Introduction

Besides being a topic of enduring fascination to laymen, deception has stimulated a vibrant field of scientific enquiry across numerous distinct research disciplines including (but by no means limited to) philosophy, psychology, economics, criminology, and in recent years, neuroscience. Despite this long, inter-disciplinary tradition, doubt appears to exist as to the possible future direction of deception research. This doubt has largely been due to discussions of the utility (or futility) of laboratory-based deception research (Vrij and Granhag, 2012). What follows is a brief general introduction to the field, highlighting key findings and an overview of the methods employed to uncover them. We hope that this will lead to an understanding of the context within which the original research (Wright et al., 2012) was formulated.

Our original paper aimed to address some of these methodological criticisms of laboratory-based research, but most significantly, to focus on the skill of the liar in addition to the lie detector. A new, socially interactive, task was developed in which participants were motivated by competition and high-value prizes. Results were analyzed using the novel application of **signal detection theory** to measure lie production ability, alongside the ability to detect lies. The main goal of the research was to assess whether skill in the production and detection of lies were correlated, that is, to assess whether good lie detectors made good liars. The results and implications of the research, most notably the description of a **“deception-general” ability** contributing to both production and detection of deception, are presented and it shall be argued that laboratory-based studies of deception are not only valid, but constitute an area ripe for theoretical development (see also Frank and Svetieva, 2012).

Deception is a ubiquitous aspect of everyday human interaction and remarkably varied in the forms it can take, the contexts in which it can occur and the motives ascribed to its perpetrators (DePaulo et al., 1996; Kashy and DePaulo, 1996). Any taxonomy of deceptive human interaction will necessarily include such diverse concepts as white lies, bluffing, lies of omission, malingering and even sarcasm (Levine, 2010). A commonly used definition of deception, which attempts to incorporate all forms of deception, is “a successful or unsuccessful deliberate attempt, without forewarning, to create in another a belief which the communicator considers to be untrue” (Vrij, 2000, p. 15). In spite of this common starting point, the ways in which deception is operationalized in the laboratory setting are still hugely varied, and almost universally problematic in some regard. It is important to note however, that central to this depiction of deception is the inherent interpersonal or social component; there is a deceiver and the deceived—the liar and the lie detector.

### Detecting deception

Whether explicitly or not, the majority of deception research has focused on lie detection. Studies have examined individual differences in lie detection ability, factors that may predict the ability of an individual to detect deception, and strategies, cues or technologies designed to improve lie detection (Vrij and Granhag, 2012). From lie detection studies such as these a number of robust results have been observed. For detailed analysis, interested readers are directed toward three comprehensive meta-analyses by DePaulo et al. (2003); Bond and DePaulo (2006, 2008).

The first of these meta-analyses (DePaulo et al., 2003) examined a range of 158 cues for their predictive utility to discriminate honest and deceptive behavior. Although several cues were found to be predictive of deception (such as response duration and vocal pitch), each was only very loosely related to deception across studies such as to be of almost no use in detecting deception given large variance in the expression of those cues within an individual, even when telling the truth, and the large degree of variance in the extent to which the cues are displayed across individuals (Levine et al., 2005). The second meta-analysis (Bond and DePaulo, 2006) focused on the accuracy of lie detection (based on a corpus of nearly 24,500 veracity judgments), and found mean “lie detection” performance to be in the region of 54% accuracy, with most studies falling within 10% of this figure (Levine, 2010). Although not much higher than may be expected by chance, this rate is nonetheless significantly different from chance. The 54% accuracy referred to above is the percentage of all statements judged which are correctly identified as truth or lie. When examined separately, accuracy at correctly identifying truths is generally higher (approximately 65%) than the rate at which lies are correctly identified as lies (approximately 40%). This “**Veracity Effect**,” is often attributed to a commonly reported response bias observed in naïve lie detectors, the so-called “**Truth Bias**.” The design of the majority of lie detection tasks usually involves 50% of stimulus items being truthful and 50% deceptive. It is observed that individuals usually identify more statements as truthful than as deceptive (around 60–65%), and therefore this response bias could account for the increased accuracy for truthful statements over deceptive ones. The inadequacies of these blunt percentage accuracy figures are discussed and a potential solution proposed in a later section describing our Signal Detection Theory analytic framework.

That no universal deception cue has yet been identified is perhaps the most broadly reported result in the literature. Similarly robust is the finding that no single individual difference measure is reliably related to deception detection accuracy when individuals perform deception detection tasks (Aamodt and Custer, 2006). The failure on behalf of behavioral psychology to identify reliable correlates of lying has prompted the application of neuroscience techniques such as electroencephalography and functional Magnetic Resonance Imaging (fMRI) in an attempt to identify a neurological signal of lying. Debate surrounding the accuracy and utility of these techniques has been fierce, but several studies have provided promising evidence for the identification of deceptive patterns of brain activity using fMRI (e.g. Hakun et al., 2009; Monteleone et al., 2009), although countermeasures can be developed which may render the technique useless in practice (Ganis et al., 2011).

### The importance of the liar

An interesting feature of the empirical literature on lie detection is that the selection of stimulus material is usually only briefly described in journal articles and examples are rarely published. The apparent assumption is that lies are invariant in quality and all lies will necessarily display some “deceptive evidence” that an accurate judge will be able to perceive and correctly attribute to attempted deception. In contrast to this view, a meta-analysis by Bond and DePaulo (2008) suggests that the outcome of any individual deceptive engagement between liar and lie detector may be more attributable to the skill (or lack thereof) of the liar than any acuity on the part of the lie detector. In spite of these data, only a small minority of studies has made any attempt to determine individual differences in the ability to lie (and tell the truth) credibly (e.g., DePaulo and Rosenthal, 1979; Riggio et al., 1987). Thus, a focus on the liar constituted one of the main aims of our original study.

### What's wrong with studying lying in the lab?

A debate rages around the ecological validity of studying deception in the lab, with some researchers arguing that lab studies are impossible to generalize to applied contexts and so therefore contribute little to our understanding of real-world deception. Criticism usually include 5 specific features of deception paradigms, including (1) the use of instructed lies, (2) the sanction of experimental lies, (3) low motivation experienced by participants, (4) low stakes for failure, and (5) limited social interaction.

#### Instructed lies

Participants recruited to act as liars or truth tellers are usually instructed to either lie or tell the truth upon experimental cues. The fact that an experimenter instructs the participant to lie or tell the truth is argued by some to remove the essence of deception by not giving the perpetrator the option to lie or tell the truth—that instead of lying, participants are merely following instructions (e.g., Kanwisher, 2009). Of course this feature of deception paradigms is not intrinsic to deception itself, and therefore experimental paradigms can, and have been, developed which remove instruction and allow participants to choose when to lie (e.g., Sip et al., 2010, 2012). The use of uninstructed paradigms introduces further problems however; including lower rate of lies produced in such situations which impacts upon statistical power and experimental control. Furthermore, allowing participants to choose when to lie is likely to introduce an experimental confound relating to confidence or strength of opinion. It is possible that participants may only choose to tell lies about issues that they can confidently lie about. These issues are likely to be those things that matter least to them, or issues for which they already have well-rehearsed lies. It has been argued that removing the choice about whether to lie means that the experimental study lacks ecological validity (Kanwisher, 2009), but we suggest that it is not always the case that in real-life we can choose when to lie and when to tell the truth. There may be many occasions where prior behavior, job or family roles, or social or moral imperatives demand deception even though the individual may not want to lie, or be confident about successfully doing so.

#### Sanctioned lies, low motivation, and low fear of failure

That lies are sanctioned in experimental settings, thereby removing the element of moral transgression, associated risk of punishment, and possible related feelings of guilt, anxiety and cognitive load (Caso et al., 2005) is a common criticism leveled at deception paradigms (Frank and Ekman, 1997). It is argued that removing the threat of punishment, which would normally accompany being uncovered as a liar, limits the availability of verbal and behavioral cues elicited by lying, in particular those cues relating to arousal and cognitive effort. In turn, it is argued that the absence of these cues contributes to the exceedingly poor rates of deception detection in experimental paradigms. Experimental evidence does not support this conjecture however; Feeley (1996) found no difference in the accuracy of judgments made by recipients of either sanctioned or unsanctioned lies, while Feeley and de Turck (1998) showed evidence that sanctioned lies were more commonly associated with behavioral cues to deception than unsanctioned lies. This result was recently replicated in our lab. Indeed, Sporer and Schwandt (2007) performed a meta-analysis of 11 studies and the only “deceptive cue” which differed as a result of sanction was smiling, in that smiles tended to increase when lies were unsanctioned.

#### Motivation

A related issue concerns the motivation to lie. It has often been argued that the low level of motivation to succeed when lying, and low fear of failure, mean that experimental studies of deception lack ecological validity. This argument is presented in two different ways—one version of the argument suggests that as the stakes for success or failure in the lab are much reduced compared to real life (as a result of sanctioned lies for example), participants may not try as hard to effect successful deceit. Conversely, it is also argued that real-life deception may be less successful than in the lab because the greater motivation to successfully deceive in real life (due to the higher stakes for success and failure) may lead to a difficulty in effecting successful deception, a so-called “Motivational Impairment Effect” (DePaulo and Kirkendol, 1989). In response to both of these criticisms we would question the assumption that all instances of deception in everyday life are of sufficient importance to cause high motivation. Indeed, observational studies suggest that most lies in everyday life are unplanned, of minimal importance and of little consequence if detected (DePaulo et al., 1996; Kashy and DePaulo, 1996). Although some researchers have argued previously that only high stakes (such as a criminal conviction) may be suitable for ecological examinations of deception, a greater awareness of the range of lies told in everyday life has led to a softening of this view (Vrij and Granhag, 2012).

#### Social interaction

In our view more worrisome than the factors discussed so far is the lack of social interaction in experimental studies of deception. Although deception is an inherently dynamic social interaction (Buller and Burgoon, 1996), only 9% of studies in a meta analysis by Bond and DePaulo (2006) featured any real interaction between the **Sender** of lies and the individuals tasked with their detection (**Receivers**). Stimulus material in lie detection tasks is usually pre-recorded (in written, video, or audio form), thus permitting limited access to potentially useful cues to deception not portrayed by these media. It is worth highlighting that without a live audience, the performance of the Sender may also be impacted since a video camera gives no feedback or sense of social contingency. In this regard, researchers examining investigative interview strategies have maintained an important aspect of everyday deception by virtue of using socially interactive paradigms (e.g. Hartwig et al., 2006). Interestingly, neuroscientists such as Sip and colleagues have frequently argued for the need for a social dimension to deception research and have implemented this within their fMRI designs investigating deception (Sip et al., 2010, 2012).

Thus, with the goal of focusing upon the liar and addressing the most theoretically relevant individual difference measure with regards to lie detection accuracy—the ability to deceive—we developed a paradigm addressing a number of the criticisms aimed at previous laboratory studies. Primarily, we hoped to address the lack of social interaction and the low motivation of participants using an interactive competitive game played for high value cash prizes. While participants were instructed whether to lie or to tell the truth on each trial, lies were directed to other participants who stood to lose if they did not detect the deception. Therefore the recipient of the lie did not sanction the lies. As detailed in the original article, we hypothesized the existence of a “deception-general ability” whereby the ability to deceive and the ability to detect deception may be related, with cognitive (Spence et al., 2004) and emotional intelligence (Sip et al., 2010) contributing to both processes. Although this review focuses on the findings from the first iteration of this novel Deceptive Interactive Task (DeceIT), the key finding of a deception-general ability has since been replicated by our group (Wright et al., submitted) and awaits examination in other labs.

## Materials and methods

### Participants

Fifty one healthy adults (27 female, mean age = 25.35 years, *SD* = 8.54) with English as a first language participated in the study. All participants provided written informed consent to participate and for data to be collected. The local Research Ethics Committee (Dept. of Psychological Sciences, Birkbeck College) granted ethical approval for the study.

### Procedure

Participants volunteered to take part in a “Communication Skills” experiment and were randomly assigned to nine groups of five participants and one group of six participants, with the constraint that group members were not previously known to each other. Participants were seated in a circle and asked to complete an “Opinion Survey” questionnaire. The questionnaire comprised 10 opinion statements (e.g., “Smoking should be banned in all public places”) to which participants responded “agree” or “disagree.” Responses to the Opinion Survey served as ground truth in the subsequent task (c.f. Mehrabian, 1971; Frank and Ekman, 2004). Participants also completed the Toronto Alexithymia Scale (TAS—Parker et al., 2001), a measure of the degree to which emotions can be identified and described in the self, and the Interpersonal Reactivity Index (IRI—Davis, 1980), a measure of empathy. These instruments provide self- and other-focused measures of emotional intelligence (Mayer et al., 1999; Parker et al., 2001). A subset of participants (*n* = 31, 61% of sample) also completed the Wechsler Abbreviated Scale of Intelligence (WAIS—Wechsler, 1999).

Participants were then informed that they were to participate in a competitive “game” against the other participants in their group that was designed to test their communication skills. They were told that two high value (£50) prizes would be awarded; one to the participant who was rated as most credible across all trials and the other to the participant who was most accurate in their judgments across all trials. Participants were required to make both truthful and dishonest statements relating to their answers on the prior “Opinion Survey,” with the objective being to appear as credible as possible regardless of whether they were telling a lie or the truth. Participants played the role of both “Communicator” (Sender) and “Judge” (Receiver), and their role changed randomly on a trial-by-trial basis, with topic being similarly randomized.

On each trial, the experimenter presented one participant with a cue card, facedown, specifying a topic from the “Opinion Survey” and an instruction to lie or tell the truth on that trial. This indicated to all participants the Sender for the upcoming trial. At a verbal instruction to “go,” the participant turned the card, read the instruction, and then spoke for approximately 20 s, presenting either their true or false opinion and some supporting argument. After the presentation of the format of the task, a practice trial was conducted for all participants and the experimenter presented a verbatim example response from the piloting phase of the study. This permitted each participant to fully preview the requirements of both Sender and Receiver roles. Following each trial, Senders were required to rate whether they thought they had been successful or unsuccessful in appearing credible using a binary “credible” or “not credible” response scale. Simultaneously, Receivers rated whether they thought the opinion given by the Sender was true or false by marking “Truth” or “Lie” on their response form. Each participant completed 10 or 20 trials as Sender, half with their true opinion and half with their false opinion. Statistical analysis demonstrated that performance did not vary as a function of the number of statements produced and so this variable is not analyzed further. The 50:50 lie-truth ratio was not highlighted to the participants at any stage to prevent strategic responding in either the Sender or Receiver roles. Following the task, participants were asked to rate the degree to which they experienced guilt, anxiety and cognitive load when lying and when telling the truth, each on a 5-point Likert scale ranging from 1 (not at all) to 5 (extremely).

### Analyzing deception detection data using signal detection theory

In deception detection experiments, researchers often wish to measure two aspects of performance: (1) the ability of receivers to discriminate lies from truths, and (2) the receiver's tendency to classify messages as true (truth bias). (1) Is typically measured as the percentage of messages that are correctly classified by the receiver (lies classified as lies and truths classified as truths). Greater than chance performance (i.e., performance that is greater than would be expected if based upon guessing) is therefore indicated by a percentage correct score greater than 50%. (2) Is most commonly indexed as the percentage of truth classifications made (across all messages); scores greater than 50% therefore indicate a general truth bias. One limitation of measuring (1) using the percentage correct measure is that, when the proportions of lie and truth messages are unequal, this measure is not independent of (2). For example, suppose that a receiver has a general tendency to classify messages as truths. If a greater proportion of true messages than false messages are presented to the receiver, then the receiver will most likely score above 50% when the percentage of correct classification score is calculated, but this will be a false impression of their detection ability: their accuracy is confounded by their truth bias. This limitation of the use of the percentage correct measure in deception research has been noted by other researchers (e.g., Bond and DePaulo, 2006). Thus, the use of the percentage correct measure is typically confined to circumstances in which there are an equal proportion of truth and lie messages, or a weighted version of the percentage correct measure is used when the proportion of lie and truth messages are unequal.

Signal detection theory is a well-established framework (Green and Swets, 1966), which can be used to provide alternative measures of detection discriminability and truth bias. Importantly, measures can be derived that are not confounded, and can be used when there are unequal proportions of lie and truth messages to be classified. In place of the percentage of correct classifications and the percentage of truth classifications, the signal detection measures of *d*′ and *C* can be used, respectively. Using this framework, a “hit” (H) can be defined as a “lie” classification to a lie message, and a “false alarm” (FA) can be defined as a “lie” classification to a truth message. *d*′ can then be calculated as the difference in the *z* transformed proportions of hits and false alarms (i.e., *d*′ = *z*(p(H)) – *z*(p(FA)). A positive *d*′ score therefore indicates a tendency to correctly distinguish lies from truths. The measure of bias, *C*, can be calculated as *C* = −0.5 × [*z*(*p*(*H*)) + *z*(*p*(*FA*))]. A negative value of *C* therefore indicates a truth bias, and a positive value of *C* indicates a bias to classify messages as lies.

### The analysis of deception production data using signal detection theory

**Figure 1:**
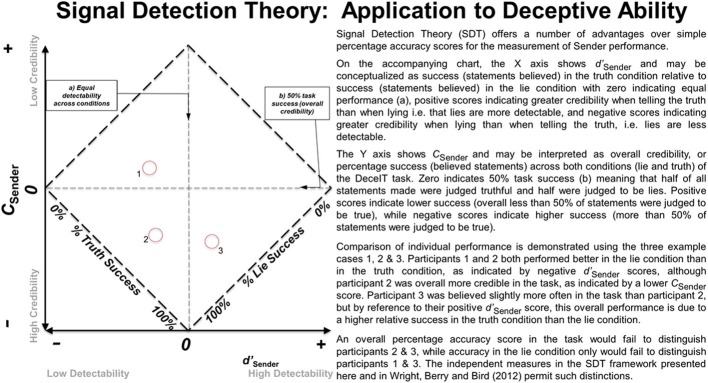
**An overview of the application of Signal Detection Theory (SDT) to the Sender role and its interpretation**.

Wright et al. (2012) showed that signal-detection measures could also be effectively applied to index the deceptive abilities of the *Senders* of truth and lie messages. Similar benefits arise from the use of independent measures of *d*′ and *C* in the Sender role as these index Discriminability (or the extent to which a Senders' lies and truth messages can be correctly distinguished) and Credibility (the extent to which a Senders' messages as a whole tend to be rated as truthful), respectively. Sender Credibility, as indexed by *C* may be thought to be analogous with the “**Demeanor Bias**” mentioned previously. Full details of the analysis strategy and the interpretation of results can be found in Wright et al. (2012) and Wright et al. (submitted). An overview of the application of SDT to deception research is provided in Figure 1. A major advantage of characterizing the performance of Receivers and Senders with the same measures (*d*′ and *C*) for each role was that it facilitated an analysis of the relationship between the measures across roles.

## Results

In line with previous studies (Caso et al., 2005) participants reported greater Guilt, Anxiety and Cognitive Load when lying than when telling the truth [statistics: Guilt *t*_(50)_ = 7.060, *p* < 0.001, *d* = 1.226, Anxiety *t*_(50)_ = 9.598, *p* < 0.001, *d* = 1.784, Cognitive Load *t*_(50)_ = 9.177, *p* < 0.001, *d* = 1.421]. Also in common with previous studies (Walczyk et al., 2003), Response Latency was significantly shorter when participants told the truth [*M* = 4.6 s, *SD* = 2.0) than when they lied (*M* = 6.5 s, *SD* = 3.1, *t*_(50)_ = −3.885, *p* < 0.001, *d* = 0.728]. Finally, task performance in the Receiver role was analyzed using conventional percentage accuracy rates and overall accuracy was found to be 54.1% (*SD* = 8.7%), not significantly different to the 54% reported previously (Levine, 2010) [*t*_(50)_ = 0.065, *p* = 0.950, *d* = 0.013] but significantly greater than chance [*t*_(50)_ = 3.335, *p* = 0.002, *d* = 0.667]. Fractional rates addressing accuracy for different types of statement showed a significantly lower mean accuracy for truths (*M* = 51.1%, *SD* = 11.9%) than for lies [*M* = 57.1%, *SD* = 10.5%, *t*_(50)_ = −3.731, *p* < 0.001, *d* = 0.746]. To compare any response bias in the Receiver role with findings from the literature, we calculated the number of statements of all types classified by Receivers as truthful and found it to be 46.7% (*SD* = 8.8%) a figure significantly lower than chance [*t*_(50)_ = −2.667, *p* = 0.005, *d* = 0.535].

**Figure 2:**
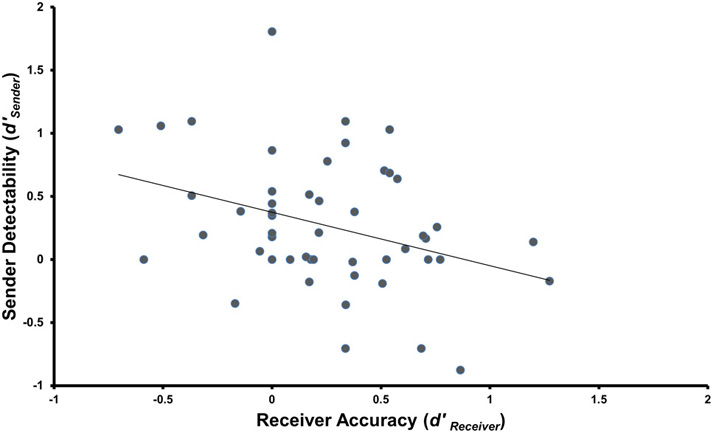
**Correlation between Sender and Receiver performance using SDT measures for Receiver Accuracy (*d*′_receiver_) and Sender Detectability (*d*′_sender_) (*r* = −0.348, *p* = 0.006, *d* = 0.742)**.

Large individual differences were observed in all of the four performance measures analyzed using Signal Detection Theory (*M d*′_receiver_ = 0.242, *SD* = 0.418; *M C*_receiver_ = −0.086, *SD* = 0.233; *M d*′_sender_ = 0.272, *SD* = 0.509; *M C*_sender_ = 0.097, *SD* = 0.256). Of principal interest is the fact that detectability in the Sender role (*d*′_*sender*_) and the ability to discriminate in the Receiver role (*d*′_receiver_) were significantly correlated (*r* = −0.348, *p* = 0.006, *d* = 0.742, see Figure 2). As the ability to discriminate truthful from deceptive messages increased, the ability to produce deceptive messages that were hard to discriminate from truthful messages increased. Interestingly, a trend was observed for decreasing detectability in the Sender role to be associated with a reduced response latency difference between truthful and deceptive statements (Spearman's *rho* = 0.259, *p* = 0.068, *post-hoc*). The only significant association with either measure of bias (Truth-Bias or Credibility) was a correlation between a Sender's confidence that they were believed and their Credibility measure, i.e., those that judged they were believed were more likely to be seen as honest independently of the veracity of their statements (Spearman's *rho* = −0.316, *p* = 0.024, *post-hoc*). Neither IQ (all *r* values < 0.184), emotional ability relating to the self (all *r* values < 0.198), nor empathy (all *r* values < 0.153) correlated with *d*′_receiver_, *C*_receiver_, *d*′_sender_, or *C*_*sender*_.

## Discussion

The relationship between the abilities to successfully produce and accurately detect deception was examined using a novel group Sender/Receiver deception task (DeceIT). This paradigm addressed widespread concerns around ecological validity in that it was socially interactive (rather than video-mediated) and sought to increase and maintain motivation in participants by introducing a competitive element with high-value monetary rewards. The reported results were comparable to patterns of results reported in the deception literature with regards to increased self-reported guilt, anxiety and cognitive load while performing the task (Caso et al., 2005), as well as the overall percentage detection accuracy rate (Levine, 2010). Furthermore, chronometric cues related to deception were replicated, whereby significantly longer response latencies were recorded for statements in which participants lied than when they told the truth about their opinion (Walczyk et al., 2005). The finding that the ability of individuals to detect deception and to successfully deceive were positively associated, is interpreted to suggest the existence of some form of “deception-general” ability, contributing to success in both roles within a deceptive encounter.

Interpreting the “deception-general” ability given the data currently available is very difficult. Deceptive skill was unrelated to IQ or self-report measures of self- or other-focused emotional intelligence. We tentatively suggest that it may be a product of practice or vigilance in everyday life through social learning (Cheney et al., 1986; Byrne, 1996) or attention to conspecifics more generally (Heyes, 2012). Speculatively, this practice may enable the individual to develop a model, likely to be implicit in nature, that they can apply in a deceptive encounter to either modulate their own behavior or to assess the behavior of another. Whether individual difference variables such as social motivation, social attention, or theory of mind contributes to the speed with which one develops a model of deceptive behavior, and therefore a deception-general ability, is at present an unanswered question. Gaining a comprehensive understanding of how people differ in their ability and propensity to deceive is an important research aim, and a present focus of our lab.

To summarize, our recent discovery of a “deception-general” ability, conferring advantage to some individuals over others in both aspects of deception, is a potentially powerful interpersonal tool delivering a competitive edge for resources and social position. We have argued that our DeceIT paradigm and Signal Detection Theory based analytic technique provide the tools to finally turn the spotlight on the liar, a variable of interest in deception research whose time was long overdue. We have shown that DeceIT addresses a number of the methodological concerns around sanction, low stakes and social interaction highlighted above. We have further suggested that lab-based research is not distinct from the vast majority of real-world deception in terms of the penalty for being caught lying, we cite evidence describing real-world lies as being frequent and often of little consequence if discovered (DePaulo et al., 1996; Kashy and DePaulo, 1996).

We have promoted the adoption of SDT techniques in future research to index the performance of both Senders and Receivers, of liars and lie detectors. Although the SDT measures initially seem less transparent than traditional percentage accuracy scores, they capture all aspects of performance relevant for deception. One can gain independent measures of an individual's skill when deceiving, their credibility or demeanor bias, their ability to detect lies, and their degree of credulity, or truth bias. These measures are confounded in traditional percentage accuracy scores. Furthermore, percentage accuracy scores, but not SDT measures, are susceptible to biases induced through factors such as the proportion of lies and truths presented to participants during the experimental task.

Certain researchers, deeply ensconced in the field, may appear somewhat frustrated or urge a wholesale change of direction in deception research. However, in keeping with the idea that it is often “darkest before the dawn,” we promote a renewed vigor in deception research, both in the lab *and* within applied settings. An important element of this drive must be the broadening of research goals beyond that of lie detection, to include lie production ability, and the mechanisms of credibility and trustworthiness that contribute to it, leveraging all the tools, insights and theoretical advancement that interdisciplinary collaboration promises.

### Conflict of interest statement

The authors declare that the research was conducted in the absence of any commercial or financial relationships that could be construed as a potential conflict of interest.

## Key Concept

Signal detection theory: An analysis technique traditionally used in psychophysics whereby the sensitivity (*d*′) and bias (*C*) of a Receiver are independently estimated. Outlined in the current paper is an approach designed to apply this technique to Sender performance offering similar benefits.
“Deception-general” ability: Refers to the recent finding that the abilities to detect and to produce deception successfully may be related. IQ and measures of self- and other-focused emotional intelligence are not correlated with this deception-general ability, further research is required to understand the contributing psychological or cognitive processes involved.
Veracity effect: The finding that when considered separately, truthful statements are more usually correctly classified as such than lies correctly identified as lies. Potentially a statistical artefact due to the Truth Bias.
Truth bias: A common response bias in lie detection tasks whereby Receivers will tend to make more “Truth” ratings than would be expected by chance. Usually lie detection experiments use an equal amount of truthful and deceptive stimuli, thus participants typically judge more than half of all stimuli as truthful.
Sender: The communicator of a message. In deception detection paradigms this constitutes the liar (or truth teller). Although Senders are by necessity part of experimental tasks, their performance is rarely reported. Such performance is argued to be critical to the outcome of a deceptive encounter.
Receivers: In deception detection tasks, the individual tasked with making the judgment will often be referred to as the Receiver. Historically, this has been the focus of interest in deception research, be it individual differences in accuracy, predictors of accuracy, or methods of training improved performance.
Demeanor Bias: Characteristics that contribute to the extent to which an individual Sender may be believed (or not) due to general appearance or communicative style.

## References

[B1] Aamodt M. G. Custer H. (2006). Who can best catch a liar? A meta-analysis of individual differences in detecting deception. Forensic Exam. 15, 6–11

[B2] Bond C. F. DePaulo B. M. (2006). Accuracy of deception judgments. Pers. Soc. Psychol. Rev. 10, 214–234 10.1207/s15327957pspr1003_2 16859438

[B3] Bond C. F. DePaulo B. M. (2008). Individual differences in judging deception: accuracy and bias. Psychol. Bull. 134, 477–492 10.1037/0033-2909.134.4.477 18605814

[B3a] Buller D. B. Burgoon J. K. (1996). Interpersonal deception theory. Commun. Theory 6, 203–242 10.1111/j.1468-2885.1996.tb00127.x

[B4] Byrne R. W. (1996). Machiavellian intelligence. Evol. Anthropol. 5, 172–180

[B5] Caso L. Gnisci A. Vrij A. Mann S. (2005). Processes underlying deception: an empirical analysis of truth and lies when manipulating the stakes. J. Invest. Psychol. 2, 195–202 10.1002/jip.32

[B6] Cheney D. Seyfarth R. M. Smuts B. B. (1986). Social relationships and social cognition in nonhuman primates. Science 234, 1361–1366 10.1126/science.3538419 3538419

[B7] Davis M. H. (1980). A multidimensional approach to individual differences in empathy. JSAS Cat. Sel. Doc. Psychol. 10, 85

[B9] DePaulo B. M. Kashy D. A. Kirkendol S. E. Wyer M. M. Epstein J. A. (1996). Lying in everyday life. J. Pers. Soc. Psychol. 70, 979–995 10.1037/0022-3514.70.5.979 8656340

[B10] DePaulo B. M. Kirkendol S. E. (1989). The motivational impairment effect in the communication of deception, in Credibility Assessment, eds Yuille J. C. (Dordrecht: Kluwer), 51–70

[B11] DePaulo B. M. Lindsay J. J. Malone B. E. Muhlenbruck L. Charlton K. Cooper H. (2003). Cues to deception. Psychol. Bull. 129, 74–118 10.1037/0033-2909.129.1.74 12555795

[B12] DePaulo B. M. Rosenthal R. (1979). Telling lies. J. Pers. Soc. Psychol. 37, 1713–1722 10.1037/0022-3514.37.10.1713 512835

[B13] Feeley T. H. (1996). Exploring sanctioned and unsanctioned lies in interpersonal deception. Commun. Res. 13, 164–173 10.1080/08824099609362083

[B14] Feeley T. H. de Turck M. A. (1998). The behavioral correlates of sanctioned and unsanctioned deceptive communication. J. Nonverbal. Behav. 22, 189–204 10.1023/A:1022966505471

[B15] Frank M. G. Ekman P. (1997). The ability to detect deceit generalizes across different types of high-stakes lies. J. Pers. Soc. Psychol. 72, 1429–1439 10.1037/0022-3514.72.6.1429 9177024

[B15a] Frank M. G. Ekman P. (2004). Appearing truthful generalizes across different deception situations. J. Pers. Soc. Psychol. 86, 486–495 10.1037/0022-3514.86.3.486 15008651

[B16] Frank M. G. Svetieva E. (2012). Eliciting cues to deception and truth: what matters are the questions asked. J. Appl. Res. Mem. Cogn. 1, 131–133 10.1016/j.jarmac.2012.04.006

[B17] Ganis G. Rosenfeld J. P. Meixner J. Kievit R. A. Schendan H. E. (2011). Lying in the scanner: covert countermeasures disrupt deception detection by functional magnetic resonance imaging. Neuroimage 55, 312–319 10.1016/j.neuroimage.2010.11.025 21111834

[B18] Green D. M. Swets J. A. (1966). Signal Detection Theory and Psychophysics. New York, NY: Wiley

[B19] Hakun J. G. Ruparel K. Seelig D. Busch E. Loughead J. W. Gur R. C. (2009). Towards clinical trials of lie detection with fmri. Soc. Neurosci. 4, 518–527 10.1080/17470910802188370 18633835

[B20] Hartwig M. Granhag P. A. Strömwall L. A. Kronkvist O. (2006). Strategic use of evidence during police interviews: when training to detect deception works. Law Hum. Behav. 30, 603–619 10.1007/s10979-006-9053-9 16977348

[B21] Heyes C. (2012). What's social about social learning? J. Comp. Psychol. 126, 193–202 10.1037/a0025180 21895355

[B22] Kanwisher N. (2009). The use of fMRI in lie detection: what has been shown and what has not, in Using Imaging to Identify Deceit: Scientific and Ethical Questions, eds Bizzi E. Hyman S. E. Raichle M. E. Kanwisher N. Phelps E. A. Morse S. J. (Cambridge, MA: American Academy of Arts and Sciences), 7–13

[B23] Kashy D. A. DePaulo B. M. (1996). Who lies? J. Pers. Soc. Psychol. 70, 1037–1051 10.1037/0022-3514.70.5.1037 8656334

[B24] Levine T. R. (2010). A few transparent liars. Commun. Yearb. 34, 1–35

[B25] Levine T. R. Feeley T. H. McCornack S. Hughes M. Harms C. (2005). Testing the effects of nonverbal behavior training on accuracy in deception detection with the inclusion of a bogus training control group. West. J. Comm. 69, 203–217 10.1080/10570310500202355

[B26] Mayer J. D. Caruso D. Salovey P. (1999). Emotional intelligence meets traditional standards for an intelligence. Intelligence 27, 267–298 10.1016/S0160-2896(99)00016-112934682

[B27] Mehrabian A. (1971). Nonverbal betrayal of feeling. J. Exp. Res. Psychol. 5, 64–73

[B28] Monteleone G. T. Phan K. L. Nusbaum H. C. Fitzgerald D. Irick J. (2009). Detection of deception using fmri: better than chance, but well below perfection. Soc. Neurosci. 4, 528–538 10.1080/17470910801903530 18633832

[B30] Parker J. D. A. Taylor G. J. Bagby R. M. (2001). The relationship between emotional intelligence and alexithymia. Pers. Indiv. Differ. 30, 107–115 10.1016/S0191-8869(00)00014-3

[B31] Riggio R. E. Tucker J. Throckmorton B. (1987). Social skills and deception ability. Pers. Soc. Psychol. 13, 568–577 10.1177/0146167287134013

[B32] Sip K. E. Lynge M. Wallentin M. McGregor W. B. Frith C. D. Roepstorff A. (2010). The production and detection of deception in an interactive game. Neuropsychologia 48, 3619–3626 10.1016/j.neuropsychologia.2010.08.013 20727906

[B33] Sip K. E. Skewes J. C. Marchant J. L. McGregor W. B. Roepstorff A. Frith C. D. (2012). What if I get busted? Deception, choice, and decision-making in social interaction. Front. Neurosci. 6:58 10.3389/fnins.2012.00058 22529772 PMC3328780

[B34] Spence S. A. Hunter M. D. Farrow T. F. D. Green R. D. Leung D. H. Hughes C. J. (2004). A cognitive neurobiological account of deception: evidence from functional neuroimaging. Philos. Trans. R. Soc. Lond. B Biol. Sci. 359, 1755–1762 10.1098/rstb.2004.1555 15590616 PMC1693447

[B34a] Sporer S. L. Schwandt B. (2007). Moderators of nonverbal indicators of deception: a meta-analytic synthesis. Psychol. Public Policy Law 13, 1–34 10.1037/1076-8971.13.1.1

[B35] Vrij A. (2000). Detecting Lies and Deceit. The Psychology of Lying and the Implications for Professional Practice. Chichester: Wiley

[B36] Vrij A. Granhag P. A. (2012). Eliciting cues to deception and truth: what matters are the questions asked. J. Appl. Res. Mem. Cogn. 1, 110–117 10.1016/j.jarmac.2012.02.004

[B37] Walczyk J. J. Roper K. S. Seemann E. Humphrey A. M. (2003). Cognitive mechanisms underlying lying to questions: response time as a cue to deception. Appl. Cogn. Psychol. 17, 755–774 10.1002/acp.914

[B37a] Walczyk J. J. Schwartz J. P. Clifton R. Adams B. Wei M. Zha P. (2005). Lying person-to-person about life events: a cognitive framework for lie detection. Pers. Psychol. 58, 141–170 10.1111/j.1744-6570.2005.00484.x

[B38] Wechsler D. (1999). Wechsler Abbreviated Scale of Intelligence (WASI). San Antonio, TX: Harcourt Assessment

[B38a] Wright G. R. T. Berry C. J. Bird G. (2012). “You can't kid a kidder”: association between production and detection of deception in an interactive deception task. Front. Hum. Neurosci. 6:87 10.3389/fnhum.2012.00087 22529790 PMC3328123

